# The Shower of Flesh and Blood

**Published:** 1841-12

**Authors:** 


### THE SHOWER OF FLESH AND BLOOD.

Our rationale of this shower, in the October number of this journal, was not satisfactory to our Lebanon friends, who declare that in adopting the rumor of the negroes' having fabricated it, we are the party "hoaxed.' The negroes it seems have not admitted their agency in concocting the shower, as we were informed they had done. Our friends, at the place where the matter was found, are confident that it must have fallen from above, and was a veritable shower. Professor Troost, of the Nashville University, visited the spot soon after the singular occurrence, and published a learned and interesting account of the circumstances in the Republican Banner. He is sure that the matter was animal, but he thinks not blood. He distinguished muscular fibres on maceration of the matter in water, which separated longitudinally, as in the case of dried beef, and were of a reddish brown color. The pieces supposed to be blood were brown and resembled glue. One piece of flesh was found an inch and a half long, emitting a very offensive smell over the field. In all the particles found there was a distinct odor of animal matter in a state of putrefaction. Dr. Troost heated both the muscular part and that which had been called blood in a glass tube, and found them affected as beef or pork would have been in the same circumstances—there was a movement in the mass, a brown fluid rose, and a black animal charcoal remained.

The history of the strange event given to Dr. Troost was, "that on Friday, August 17, between one and two o'clock, P. M., the negroes of Mr. Chandler, near Lebanon, came in and reported that it had been raining blood in the tobacco field where they had been at work; that near noon there was a rattling noise like rain or hail, and drops of blood, as they supposed, that fell from a red cloud which was flying over. Intelligent men visited the ground, and observed drops of blood on the upper surface of the tobacco leaves, and portions of flesh and fat emitting a very offensive odor over the field." Dr. Troost cites many instances of red rain, red dust, red sand, red snow, showers of blood, so called, &c., in various centuries from 472 of our era to 1814, with the authorities, and concludes with a theory for this singular precipitate which we subjoin. After alluding to the well known power of the wind to transport solid materials through the atmosphere over a wide space, and to a great height, he observes: "Such a wind might have taken up part of an animal which was in a state of decomposition, and have brought it in contact with an electric cloud, in which it was kept in a state of partial fluidity or viscosity. In this case the cloud which was seen by the negroes, as well as the state in which the material were found is accounted for."

Supposing the facts to be as stated to Prof. Troost, this is a highly probable conjecture. The power of whirlwinds to elevate and transport solid and heavy bodies and deposite them in distant places, is a matter of familiar observation. Fish have been raised in this manner high into the air, and have fallen again in showers. The only matter about which we have had doubts was the fact of the descent of this flesh and blood from above. Y.

